# Impact of 5-fluorouracil metabolizing enzymes on chemotherapy in patients with resectable colorectal cancer

**DOI:** 10.3892/or.2014.3299

**Published:** 2014-07-02

**Authors:** TAKUMI OCHIAI, MASAHIKO UMEKI, HIROSHI MIYAKE, TATSUMI IIDA, MINORU OKUMURA, KAZUHIDE OHNO, MASASHI SAKAMOTO, NOBUKAZU MIYOSHI, MASAHIKO TAKAHASHI, HIDENORI TSUMURA, YUKIHIKO TOKUNAGA, HARUHIKO NAITOU, TAKUJI FUKUI

**Affiliations:** 1 Department of Surgery, Tobu Chiiki Hospital, Tokyo Metropolitan Health and Medical Treatment Corporation, Tokyo 125-8512, Japan; 2 Department of Surgery, Hyogo Prefectural Awaji Medical Center, Hyogo 656-0021, Japan; 3 Department of Surgery, Kasukabe Municipal Hospital, Saitama 344-0067, Japan; 4 Department of Surgery, Nishimino Kosei Hospital, Gifu 503-1316, Japan; 5 Department of Surgery, Hitachi General Hospital, Ibaragi 317-0077, Japan; 6 Department of Surgery, Matsudo City Hospital, Chiba 271-0064, Japan; 7 Department of Surgery, Mitsui Memorial Hospital, Tokyo 101-8643, Japan; 8 Department of Surgery, Kure Kyosai Hospital, Hiroshima 737-0811, Japan; 9 Department of Surgery, Hokkaido P.W.F.A.C Asahikawa-Kosei General Hospital, Hokkaido 078-8211, Japan; 10 Department of Surgery, Koshigaya Municipal Hospital, Saitama 343-0023, Japan; 11 Department of Surgery, Japanpost Osaka-kita Teishin Hospital, Osaka 530-0016, Japan; 12 Department of Surgery, Hokkaido Cancer Center, Hokkaido 003-0804, Japan; 13 Department of Surgery, Kariya Toyota General Hospital, Aichi 448-8505, Japan

**Keywords:** 5-fluorouracil, orotate phosphoribosyltransferase, dihydropyrimidine dehydrogenase, adjuvant chemotherapy, colorectal cancer

## Abstract

Although 5-fluorouracil (5-FU) is an important drug for colorectal cancer (CRC) treatment, no useful biomarker is currently available to predict treatment response. Since 5-FU is converted into active or inactive forms by orotate phosphoribosyltransferase (OPRT) or dihydropyrimidine dehydrogenase (DPD), a correlation between these enzymes and response to 5-FU has been suggested. However, such a correlation has not been investigated prospectively. Therefore, in the present study, we aimed to prospectively evaluate whether OPRT and DPD were predictive factors of the response to 5-FU treatment in patients with resectable CRC. The present investigation was designed as a multicenter prospective cohort study. OPRT and DPD activities were assessed in biopsy samples, obtained surgically from patients with resectable CRC. The OPRT/DPD ratio was calculated and the cut-off values for this ratio were determined for 5-year disease-free survival (DFS) and overall survival (OS). Patients were treated with 5-FU/leucovorin (LV) regimens and oral 5-FU. The endpoint of this study was the correlation between the OPRT/DPD ratio and 5-year DFS and OS. The cut-off value for the OPRT/DPD ratio was determined by using the maximum χ^2^ statistic method against 5-year DFS and OS. Sixty-eight patients were enrolled from July 2003 to May 2005. The median follow-up period was 1925 days. The OPRT/DPD ratio cut-off values for 5-year DFS and OS were 0.015 and 0.013, respectively. During the 5-year DFS and OS periods, patients with higher cut-off values had a better prognosis than those with lower ratios (P=0.03 and 0.02, respectively). In conclusion, our results suggest that the OPRT/DPD ratio could be a predictive factor for response to 5-FU/LV adjuvant chemotherapy.

## Introduction

Various useful biomarkers for molecular targeted drugs have recently been identified in advanced gastrointestinal cancer chemotherapy. For example, human epidermal growth factor receptor 2 (HER2) is involved in the pathogenesis and poor outcomes of several cancer types, including advanced gastric cancer. Molecular targeted drugs, such as trastuzumab, have been shown to prolong overall survival and progression-free survival in HER2-positive gastric cancer (1). In colorectal cancer (CRC) chemotherapy, the Ras status is a very useful biomarker for cetuximab and panitumumab treatment (2–13). The discovery of such biomarkers has led to the advancement of personalized medicine.

5-Fluorouracil (5-FU) is commonly used worldwide for the treatment of various tumors and is a key drug in CRC treatment. However, no useful biomarker is available to predict tumor response to 5-FU treatment. Consequently, individualized therapy with 5-FU is not available, despite extensive investigation on the correlation between 5-FU metabolic enzymes and antitumor effects to predict drug efficacy. Orotate phosphoribosyltransferase (OPRT) mostly converts 5-FU into its active form in the first metabolizing step, whereas dihydropyrimidine dehydrogenase (DPD) is the initial enzyme of 5-FU catabolism. Additionally, these enzymes also convert 5-FU pro-drugs such as S-1 and capecitabine into their active or inactive forms. Therefore, it is important to evaluate the activities of these enzymes to better understand the antitumor effects of 5-FU. Although there are several reports on the correlation between these enzymes and response to 5-FU (14–23), such a correlation has not been prospectively investigated in the adjuvant chemotherapy setting.

In the present study, we aimed to prospectively evaluate whether OPRT and DPD are predictive factors of 5-FU response in patients with resectable CRC.

## Patients and methods

### Study population

Patients were enrolled in the present study based on the following eligibility criteria: age of 18–75 years, histologically confirmed colon adenocarcinoma after curative surgery, Dukes’ clinical staging of B or C, Eastern Cooperative Oncology Group performance status of 0–1, no prior chemotherapy or radiotherapy, sufficient oral intake capability, and adequate major organ function. Other exclusion criteria were the presence of continuous double cancers and asynchronous multiple cancers.

### Study design and treatment

The present investigation was designed as a multicenter prospective cohort study, involving collaborative efforts from 13 centers. All patients provided written informed consent. The institutional review board or independent ethics committees approved the study protocol at each center. The endpoint of this study was the correlation between OPRT/DPD activity ratio and 5-year disease-free survival (DFS) and overall survival (OS). All patients underwent complete CRC resection without preoperative chemotherapy. They subsequently received adjuvant chemotherapy with 5-FU/leucovorin (LV) regimens as follows: bolus 5-FU (333 mg/m^2^) and a 2-h infusion of LV (167 mg/m^2^) on day 1 weekly for 6 consecutive weeks postoperatively. Thereafter, the patients were treated with oral 5-FU tablets (200 mg/day) for 2 years. This treatment was changed to other chemotherapy regimens when cancer recurrence was confirmed.

### OPRT activity

A tissue sample weighing ~300 mg was obtained from each resected tumor and immediately frozen and stored at −80°C until radioassay for OPRT activity determination (24). Briefly, tissue samples were homogenized and centrifuged to obtain the supernatant. The supernatant was then mixed with an equal volume of a substrate solution containing 100 mM Tris-HCl (pH 7.5), 100 mM MgCl_2_, 10 mM phosphoribosyl pyrophosphate, 30 mM 2-glycerophosphate, 1.6 mM α,β-methylene adenosine diphosphate, and 8 mM [^3^H]-5-FU. The reaction was stopped at 0, 5, 10, and 15 min after the addition of the substrate solution. The reaction mixture was then centrifuged to remove unreacted [^3^H]-5-FU. The reaction rate per minute was calculated by measuring the production of 5-fluorouridine monophosphate over time using a liquid scintillation counter.

### DPD activity

A tissue sample weighing ~300 mg was obtained from the resected tumor and immediately frozen and stored until radioassay for DPD activity determination (25,26). DPD activity was calculated by measuring the total production of metabolites after [^14^C]-5-FU was added to the homogenized tissue sample. These metabolites included dihydrofluorouracil, 2-fluoro-β-ureidopropionate, and α-fluoro-β-alanine. 5-FU and the metabolites were separated by thin-layer chromatography.

### Statistical analysis

The cut-off values for the OPRT/DPD ratio against recurrence and death were calculated by using the maximal χ^2^ statistic method (27,28). Subsequently, patients were classified according to their OPRT/DPD ratios into a group with ratios less than or equal to the cut-off value and another group with ratios above this value. The Pearson’s χ^2^ test was used to compare the recurrence and death rates of the two cohorts. The OPRT/DPD ratio that yielded the largest χ^2^ test result was selected as the optimal cut-off value. The DFS and OS rates according to cut-off values of OPRT and DPD activities were determined using Kaplan-Meier analysis. The survival curves of the two cohorts, categorized by cut-off values, were compared by the log-rank test. Cox’s regression method was used for the multivariate analysis of prognostic factors for DFS and OS. Data were analyzed using the Statistical Package for the Social Sciences (SPSS) for Windows version 21 (SPSS Inc., Chicago, IL, USA). A P-value <0.05 was regarded as statistically significant.

## Results

### Patients

Sixty-eight patients were enrolled from July 2003 to May 2005. Patient characteristics are summarized in Table I. The median follow-up period was 1925 days.

### Enzyme activity

The enzyme activities and OPRT/DPD ratio are shown in Table II.

### Cut-off values

The cut-off values for the OPRT/DPD ratio against DFS and OS obtained by maximal χ^2^ statistics were 0.01467 (χ^2^ value =7.863) and 0.01254 (χ^2^ value =8.05), respectively.

### Correlation between OPRT/DPD ratio and DFS

**Figure 1:**
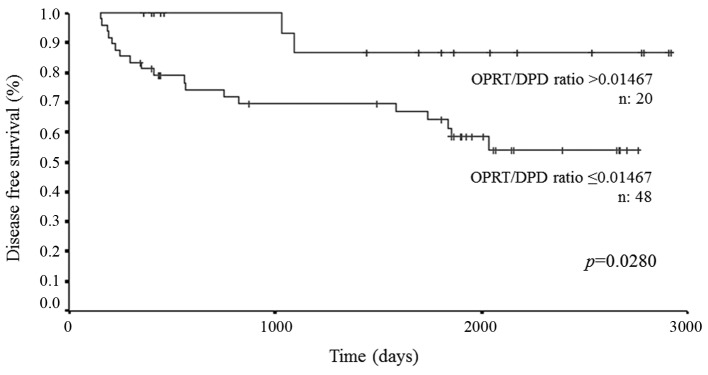
Disease-free survival rates for the two cohorts including patients with OPRT/DPD ratios less than and equal to the cut-off values.

Patients were divided into high and low OPRT/DPD ratio cohorts using a cut-off value of 0.01467. No significant differences in patient characteristics were observed between the two cohorts (Table III). The DFS rates for each cohort are shown in Fig. 1. Patients in the high OPRT/DPD ratio cohort had a significantly better prognosis for DFS than those in the low OPRT/DPD ratio cohort (P=0.0280). In addition, a high OPRT/DPD ratio [hazard ratio (HR), 0.85; 95% confidence interval (CI), 0.011–0.664; P=0.019] and node status (HR, 2.278; 95% CI, 1.175–4.416; P=0.015) were identified as independent predictive factors for better DFS by multivariate analysis (Table IV).

### Correlation between OPRT/DPD ratio and OS

**Figure 2:**
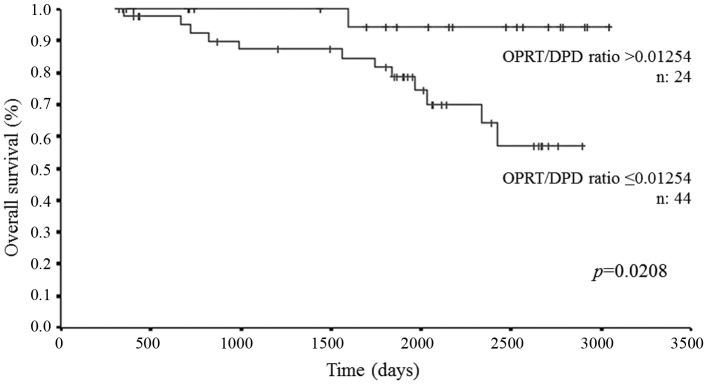
Overall survival rates for the two cohorts including patients with OPRT/DPD ratios less than and equal to the cut-off values

Patients were divided into high and low OPRT/DPD ratio cohorts using a cut-off value of 0.01254. No significant differences in patient characteristics were observed between the two cohorts (Table V). The OS rates for each cohort are shown in Fig. 2. Patients in the high OPRT/DPD ratio cohort had a significantly better prognosis for OS than those in the low OPRT/DPD ratio cohort (P=0.0208). Furthermore, a high OPRT/DPD ratio (HR, 0.112; 95% CI, 0.014–0.911; P=0.041) was identified as an independent predictive factor for better OS by multivariate analysis (Table VI).

## Discussion

OPRT is involved in the conversion of 5-FU to the active nucleotide and is considered to be a key enzyme in the first metabolizing step, leading to DNA synthesis inhibition and RNA dysfunction. DPD is the initial enzyme of 5-FU catabolism. Thus, OPRT and DPD are believed to be essentially associated with the antitumor effect of 5-FU (14–23). In the present cohort study, we prospectively evaluated the correlation between tumor OPRT/DPD ratio and response to 5-FU in CRC patients receiving 5-FU-based adjuvant chemotherapy.

We found that patients in the high OPRT/DPD ratio cohort had significantly better DFS and OS than those in the low ratio cohort. These results were consistent with the findings that 5-FU could be more easily metabolized to fluorodeoxyuridine monophosphate in the high OPRT/DPD ratio cohort, thus resulting in the induction of higher antitumor effects by 5-FU.

The nodal status and tumor OPRT/DPD ratio were identified as significant predictive factors for DFS by multivariate analysis in the present prospective study. The identification of nodal status as a predictive factor was quite logical. However, only the OPRT/DPD ratio was identified as a significant independent predictive factor for OS by multivariate analysis. The reason for this finding may be due to the influence of treatment with multiple drug combinations (such as irinotecan, oxaliplatin, molecularly targeted drugs) in addition to 5-FU after disease recurrence.

For all cancer types, the primary purpose of adjuvant chemotherapy is to eliminate traces of residual disease, which are likely to exist in high-risk patients. This prospective study revealed that the tumor OPRT/DPD ratio could be a useful independent factor to select high-risk patients for adjuvant chemotherapy. Thus, the OPRT/DPD ratio could be a predictive factor for 5-FU-based adjuvant chemotherapy. Patients in the low OPRT/DPD ratio cohort had significantly worse DFS and OS. A low OPRT/DPD ratio could be due to low OPRT activity or high DPD activity. In patients with low OPRT activity, the antitumor effects of 5-FU were not expected. Therefore, adjuvant chemotherapy with multiple drug combination (leucovorin and fluorouracil plus oxaliplatin; FOLFOX) was recommended for low OPRT activity patient. In high DPD activity patient, on the other hand, 5-FU involving a DPD inhibitor was recommended (e.g. S-1, leucovorin and S-1 plus oxaliplatin; SOX). S-1 involves gimeracil to inhibit DPD, therefore, the serum 5-FU level is kept high. Recently, the safety profile of S-1 and SOX was disclosed with adjuvant chemotherapy trials for colorectal cancer (29). In advanced CRC chemotherapy, moreover, there are several reports concerning the efficacy of SOX (30,31).

There were several limitations to this prospective study. First, the sample size was small. A larger sample size would have improved the quality of the data. Second, in the present study, 5-FU metabolic enzymes were evaluated in tumor tissue instead of cancer cells. Although 5-FU metabolic enzymes in cancer cells have been previously investigated using the microdissection method, cancerous tissue includes not only cancer cells but also stromal cells such as cancer-associated fibroblasts, tumor endothelial cells, tumor associated macrophages, and many other cells present in the tumor microenvironment. Recently, the tumor microenvironment has been reported to play an extremely important role in the progression of cancer, which suggests that cancer stromal cells contribute to cancer progression (32–38). Nagano *et al* reported that the DPD mRNA level in cancer stromal tissues was significantly higher than that in cancer cells (39). Since tumor tissue, including cancer stromal cells, was evaluated in the present study, our findings may be extremely important.

In conclusion, our results suggest that patients with a high OPRT/DPD ratio had significantly better DFS and OS than those with a low OPRT/DPD ratio. Moreover, the tumor OPRT/DPD ratio was identified as a significant predictive factor for DFS and OS by multivariate analysis. These results suggest that the tumor OPRT/DPD ratio may be a predictive factor of 5-FU response in patients with resectable CRC. In addition, the tumor OPRT/DPD ratio may contribute to the individualization of 5-FU-based chemotherapy in the clinical setting.

## Figures and Tables

**Table I:** Demography and baseline patient characteristics.

Variables	Value
Number of patients (n)	68
Age in years [mean (range)]	66 (35–75)
Gender (male/female)	42/26
ECOG PS (0/1/unknown)	58/4/6
Primary cancer site (colon/rectum/unknown)	40/24/4
Dukes’ stage (B/C/unknown)	3/62/3

ECOG PS, Eastern Cooperative Oncology Group performance status.

**Table II:** Activity of OPRT and DPD.

Variables	Mean	Range
OPRT (nmol/min/mg protein)	0.270	0.034–0.712
DPD (pmol/min/mg protein)	36.5	6.0–156.0
OPRT/DPD ratio	0.01360	0.00082–0.057

OPRT, orotate phosphoribosyltransferase; DPD, dihydropyrimidine dehydrogenase.

**Table III:** Patient characteristics for disease-free survival.

Variables	≤ Cut-off value	> Cut-off value	P-value
Mean age, years	65.5	66.0	0.929
Gender (male/female)	29/19	13/7	0.723
ECOG PS (0/1)	42/3	16/1	0.911
Primary cancer site (colon/rectum)	30/16	10/8	0.473
pN (0/1/2/3)^a^	1/38/6/2	2/10/5/1	0.159
Dukes’ stage (B/C)	1/46	2/16	0.183

Cut-off value = 0.01467.

a pN, according to the Japanese classification of colorectal cancer (6^th^ edition): pN0, no regional lymph node metastasis histologically; pN1, pN2, pN3, increasing involvement of regional lymph nodes histologically.

ECOG PS, Eastern Cooperative Oncology Group performance status.

**Table IV:** Multivariate analysis of disease-free survival.

Variables	Hazard ratio	95% CI	P-value
Cut-off value (≤0.01467/>0.01467)	0.85	0.011–0.664	0.019
Age in years	1.013	0.957–1.072	0.679
Gender (male/female)	0.519	0.193–1.400	0.195
ECOG PS (0/1)	0.573	0.072–4.544	0.598
Primary cancer site (colon/rectum)	2.014	0.633–6.405	0.236
pN (0/1/2/3)^a^	2.278	1.175–4.416	0.015

a pN, according to the Japanese classification of colorectal cancer (6^th^ edition): pN0, no regional lymph node metastasis histologically; pN1, pN2, pN3, increasing involvement of regional lymph nodes histologically.

CI, confidence interval; ECOG PS, Eastern Cooperative Oncology Group performance status.

**Table V:** Patient characteristics for overall survival.

Variables	≤ Cut-off value	> Cut-off value	P-value
Mean age, years	67.0	64.5	0.682
Gender (male/female)	28/16	14/10	0.667
ECOG PS (0/1)	38/3	20/1	1.00
Primary cancer site (colon/rectum)	27/15	13/9	0.683
pN (0/1/2/3)^a^	1/34/6/2	2/14/5/1	0.462
Dukes’ stage (B/C)	1/42	2/20	0.263

Cut-off value = 0.01254.

a pN, according to the Japanese classification of colorectal cancer (6^th^ edition): pN0, no regional lymph node metastasis histologically; pN1, pN2, pN3, increasing involvement of regional lymph nodes histologically.

ECOG PS, Eastern Cooperative Oncology Group performance status.

**Table VI:** Multivariate analysis of overall survival.

Variables	Hazard ratio	95% CI	P-value
Cut-off value (≤0.01254/>0.01254)	0.112	0.014–0.911	0.041
Age in years	1.009	0.940–1.083	0.813
Gender (male/female)	1.568	0.465–5.293	0.468
ECOG PS (0/1)	2.350	0.254–21.716	0.452
Primary cancer site (colon/rectum)	1.308	0.306–5.584	0.717
pN (0/1/2/3)^a^	1.682	0.707–4.001	0.240

pN^a^, according to the Japanese classification of colorectal cancer (6th edition): pN0, no regional lymph node metastasis histologically; pN1, pN2, pN3, increasing involvement of regional lymph nodes histologically. CI, confidence interval; ECOG PS, Eastern Cooperative Oncology Group performance status.
